# A multiple modulation synthesis method with high spatial resolution for noninvasive neurostimulation

**DOI:** 10.1371/journal.pone.0218293

**Published:** 2019-06-20

**Authors:** Qiaoqin Xiao, Zhenyu Zhong, Xiaozheng Lai, Huabiao Qin

**Affiliations:** 1 School of Electronic and Information Engineering, South China University of Technology, Guangzhou, Guangdong, China; 2 School of Computer Science and Engineering, South China University of Technology, Guangzhou, Guangdong, China; Lanzhou University of Technology, CHINA

## Abstract

Noninvasive neurostimulation plays a pivotal role in the direct control of neural circuits and the modulation of neuronal function. However, it is difficult to balance both spatial resolution and penetration depth when stimulating deep neurons. Here, we designed a multiple (time-division, frequency and polarity) modulation synthesis (MMS) method for noninvasively stimulating deep neurons with low-frequency envelopes. Compared to conventional transcranial electrical stimulation, we demonstrated that it can stimulate deep neurons at the desired firing rate (beat frequency) with higher spatial resolution via a computational model combining finite element analysis and Hodgkin-Huxley action potential model. Additionally, we measured the distribution of stimulus waveforms in saline solution to validate its effect. Taken together, the results of this study indicate that MMS stimulation with higher spatial resolution is steerable and might be a potential alternative to traditional implanted electrodes.

## Introduction

Electrical stimulation has been widely used in therapeutic applications, including the treatment of neurological and psychiatric disorders, through modulating neuronal functions with high spatial and temporal resolution. Nevertheless, deep brain stimulation with implanted electrodes is accompanied by adverse effects such as infection, limited longevity of electrical components, and requirement of battery replacement [1]. In addition, implanted electrodes are costly and surgery is expensive.

Noninvasive stimulation, such as transcranial magnetic stimulation (TMS) [2], transcranial alternating current stimulation (tACS), and transcranial direct current stimulation (tDCS) [3], produce pulsed magnetic fields or pulsed electric fields to modulate excitability of neurons by placing electrodes on the scalp. The spatial distribution of electric fields is applied to determine the neuronal modulation at each location and spatial resolution [3]. Deeper neurons can be stimulated effectively with higher stimulus current. However, the diffusion of the electric field induces unintended excitation of other neurons and reduces the accuracy of electrical stimulation. There is a trade-off between penetration depth and spatial resolution. Additionally, the high amplitudes that are required to activate deep structures might damage tissue, electrodes and dermal layers. Both TMS and transcranial electrical stimulation (TES) have relatively lower spatial precision (cm-level) compared to invasive methods (mm-level) [4]. Additionally, transcranial focused ultrasound stimulation (tFUS) [5] is more focused than both TMS and TES as its effects are expressed in mm-level. However, its mechanism of action is not clear and may damage other tissues.

Recently, temporally interfering (TI) electric fields [6] have been investigated in the mouse, and the study draws the conclusion that interferential current can stimulate the deep-lying hippocampus without the recruitment of overlying cortex. In deeper regions, temporally interfering electric fields induce low-frequency envelopes, which have enough relaxation periods when the superposition waveforms are below the current threshold to overcome high-frequency fatigue of neurons. At the same time, kilohertz-frequency alternating currents, such as burst-modulated and premodulated interferential alternating currents [7], can produce depth-efficient stimulation of nerves and muscle due to high penetration of kilohertz-frequency in human tissue. Intersectional short pulse (ISP) stimulation [8] achieves focal stimulation via spatiotemporal multiplexing. However, the spatial precision of TI and ISP stimulation has not been qualified and needs to be improved via multiple sets of electric fields.

On the basis of TI stimulation and computational models, we introduce a novel noninvasive method that stimulates the desired neurons at depth with high spatial resolution. Utilizing multiple electrodes on the scalp and applying multiple (time-division, frequency and polarity) modulation synthesis (MMS) stimulus waveforms, we can control the excitable region of deep neurons at the desired firing rate with higher spatial resolution.

The computational model is a combination of electric fields and neuronal action potentials. We utilize finite element method (FEM) to calculate the distribution of stimulus waveforms in the multi-layered concentric brain model. Additionally, somas are modeled in NEURON software to evaluate neuronal excitability across the whole brain. Integrating FEM and NEURON, we conclude that our noninvasive method can effectively stimulate deep neurons at the desired firing rate (beat frequency) with higher spatial resolution compared to TI stimulation. Finally, we measure the distribution of stimulus waveforms in saline solution to validate that the superposition of waveforms is consistent with the simulation in FEM. The MMS method for neurostimulation with high spatial resolution might be a potential alternative to traditional implanted electrodes in deep brain stimulation.

## Materials and methods

### Multiple (time-division, frequency and polarity) modulation synthesis (MMS)

**Fig 1:**
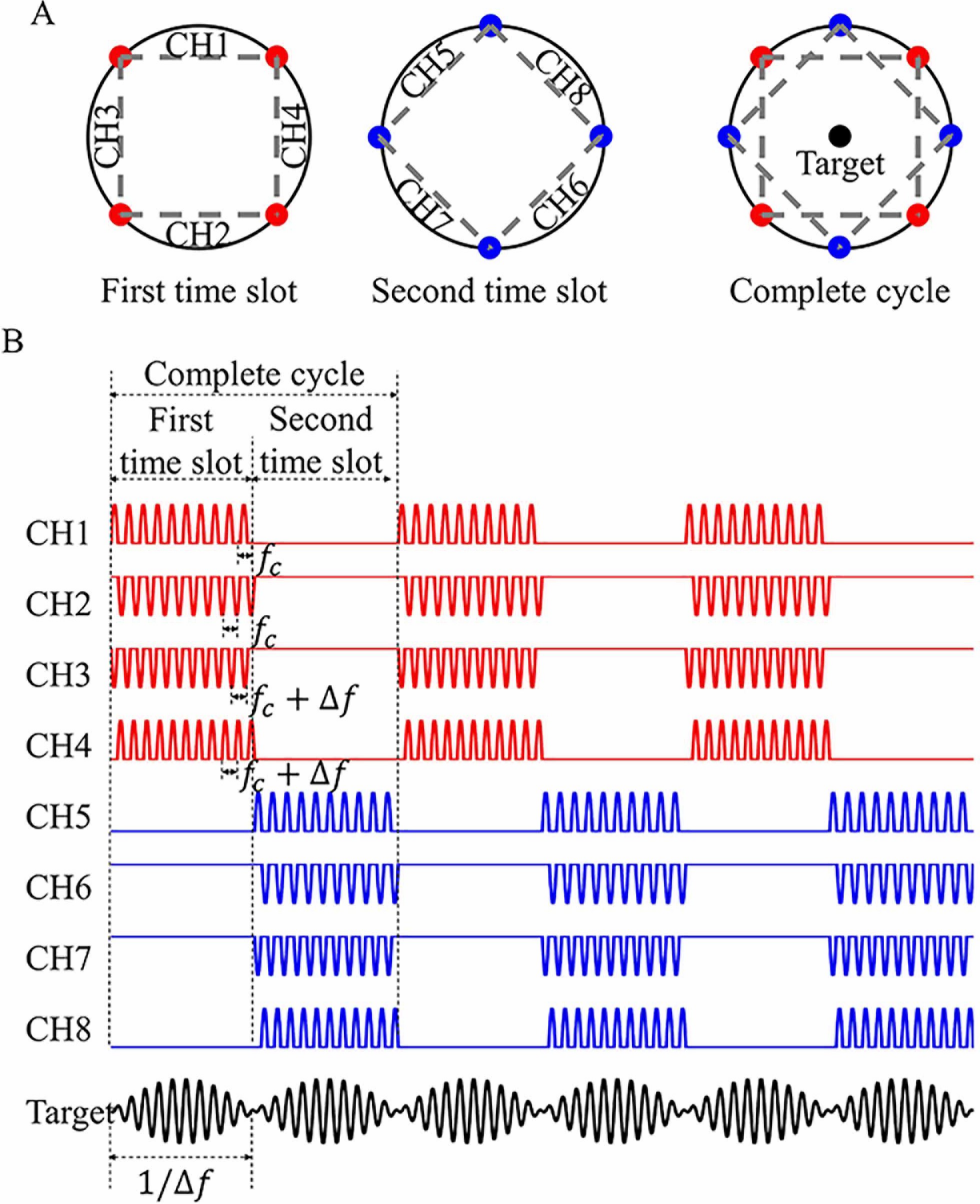
Conceptual illustration of multiple (time-division, frequency and polarity) modulation synthesis (MMS). (A) Arrangement of multiple electrodes acting in the first time slot (red), second time slot (blue). Target: the desired target region. (B) Output waveforms from various channels (CH1, CH2, …, CH8) and their superposition with the same amplitude (Target). CHs 1–8 are applied with positive and negative aspects of sine wave with different high carrier frequencies of *f*_*c*_ and *f*_*c*_+Δ*f* (*f*_*c*_ = 2 kHz, Δ*f* = 100 Hz), respectively. Each of the four channels reproduces low-frequency (Δ*f*) envelope in their respective time slots. Please note that the length of kilohertz-frequency wave is shown disproportionally for better visibility.

The target region (Target) in response to stimulus waveforms with low-frequency (Δ*f*) standard envelopes and high carrier frequency (*f*_*c*_) is shown in Fig 1. The stimulus waveforms were superposition of multiple channels (CHs 1–8) with different frequency of Δ*f* based on MMS. CH 1 and CH 3 output positive aspects of sine wave with frequencies of *f*_*c*_ and *f*_*c*_+Δ*f*, respectively. CH 2 and CH 4 output negative aspects of sine wave with frequencies of *f*_*c*_ and *f*_*c*_+Δ*f*, respectively. CHs 1–4 reproduced one part of low-frequency envelopes during the first time slot. Similarly, CHs 5–8 reproduced the other part of envelopes during the second time slot. In the subsequent analysis, we selected beat frequency (Δ*f*) of 100 Hz, carrier frequency (*f*_*c*_) of 2 kHz, a time slot of 40 ms and a complete cycle of 80 ms. Eight channels brought various spatial weightings of current densities across volume conductor respectively. Temporal summation and different spatial weightings of each channel produced different stimulus waveforms. Only in the target region, where current densities by all eight channels were equal in amplitude, the stimulus waveforms were standard, low-frequency envelopes and elicited the most neuronal excitability in a complete cycle. Otherwise, the current densities from the eight channels were combined with different spatial weightings and their summation was non-standard envelope.

### FEM with multi-layered brain model and multiple electrodes

**Fig 2:**
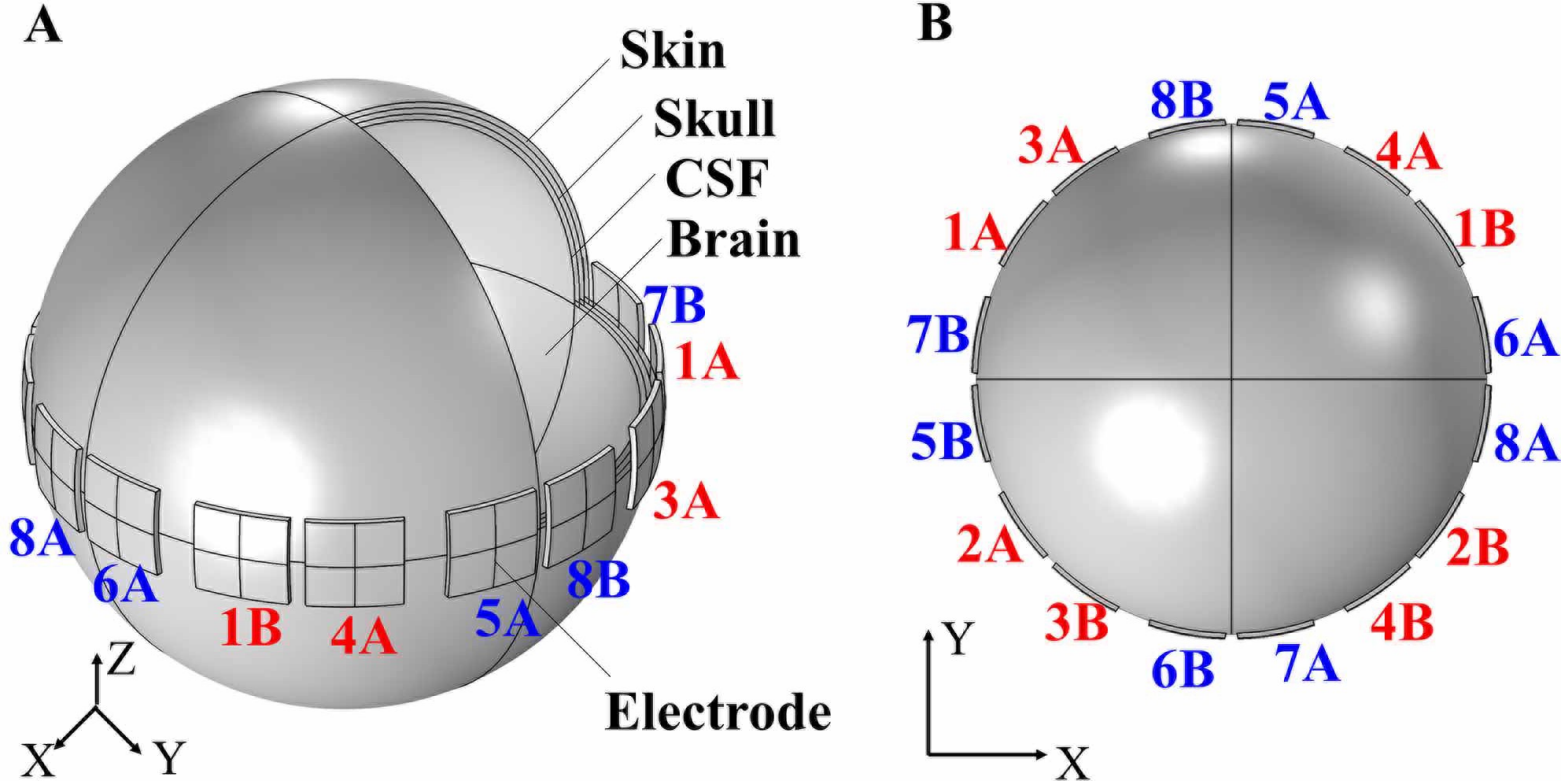
Multi-layered concentric model for noninvasive deep brain stimulation. (A) Three-dimensional view of electrodes and head model, including skin, skull, CSF and brain. (B) Cross-sectional view of electrodes arrangement. Electrodes 1A, 2A, …, 8A represent output electrode, electrodes 1B, 2B, …, 8B represent return electrode. Each channel (CH1, CH2, …, CH8) consists of an output and a return electrode. For example, channel CH1 connects electrode 1A (output electrode) and 1B (return electrode).

As shown in Fig 2, the simplified multi-layered head model and multiple electrodes were created in COMSOL Multiphysics version 4.3 (COMSOL, Stockholm, Sweden) to calculate induced current densities generated by each set of electrodes respectively, based on finite element method (FEM). The brain model was simplified into concentric spheres comprised of four layers: scalp, skull, cerebrospinal fluid (CSF) and brain [9]. Table 1 lists the size, relative permittivity *ε*_*r*_ and conductivity *σ* of the above tissue medium [10]. Sixteen electrodes, with a size of 25 mm × 25 mm, thickness of 2 mm, azimuthal spacing of 37.5 mm and material of copper, were placed around scalp surface. Each set of electrodes was driven with voltage-source terminal and ground boundary conditions. The FEM utilized relative tolerance of 0.001 for convergence criteria, 189069 tetrahedral elements and linear shape function.

**Table 1:** Geometrical dimensions and dielectric properties of tissue at 2 kHz frequency.

	Relative permittivity (*ε*_*r*_)	Conductivity σ (S/m)	Radial thickness (mm)
Scalp	31034	0.0008	2
Skull	1700	0.0202	2
CSF	109	2	2
Brain	94300	0.1230	94

Instead of stationary solution for direct current, we used frequency-domain solution to calculate various current densities generated by each set of electrodes in the brain model. The frequency-domain solution was based on Maxwell’s equations:

$$\vec{D}=\varepsilon_{0}\varepsilon_{r}\vec{E}$$ (1)

$$\vec{J}=\sigma \vec{E}+j\omega \vec{D}=\sigma \vec{E}+j\omega \varepsilon_{0}\varepsilon_{r}\vec{E}$$ (2)

$$\nabla ∙\vec{J}=-\nabla ∙[(\sigma +j\omega \varepsilon_{0}\varepsilon_{r})\nabla V]=-\frac{\partial \rho }{\partial t}$$ (3)

where $\vec{D},\;\vec{E}$ and $\vec{J}$ represent displacement field, electric field and electric current density respectively, ω represents frequency, ε_r_ represents relative permittivity, $\frac{\partial \rho }{\partial t}$ represents the charge build up with time at the same point, ∇∙ represents the divergence of a vector function and ∇ represents a scalar function. From Eq 2, the former is conduct current $\vec{J_{C}}$ and the latter is displacement current $\vec{J_{D}}$. Owing to high relative permittivity of tissue (see Table 1), the displacement current $\vec{J_{D}}$ with kilohertz frequency should not be ignored [11]. Therefore, with the same applied voltage, the current density $\vec{J}$ generated by kilohertz-frequency alternating current (AC) in deep tissue was larger than low-frequency AC or direct current. In summary, kilohertz-frequency AC had a good penetration across tissue medium due to its higher displacement current corresponding to lower capacitive resistance ($X=\frac{1}{2\pi fC}$) in tissue medium.

### Modeling of action potentials

We used the well-known Hodgkin-Huxley (H-H) model [12] to establish single-compartment neurons and evaluate the effect of specific stimulus waveforms as a current injected into a soma. The H-H model considered three fundamental active membrane channels (Na^+^, K^+^ and leakage channel) and consisted of four differential equations.

$$\frac{dv}{dt}=-\frac{1}{C_{m}}(I_{Na}+I_{k}+I_{leak}-I_{app})$$ (4)

$$I_{{Na}^{+}}={\bar{g}}_{Na}m^{3}h(v-E_{Na})$$ (5)

$$I_{K^{+}}={\bar{g}}_{K}n^{4}h(v-E_{K})$$ (6)

$$I_{leak}=g_{l}(v-E_{leak})$$ (7)

All gating variables were voltage dependent and given by following equations:

$$\alpha_{m}=\frac{0.1(v+45)}{1-\exp\left(-\frac{v+45}{10}\right)},\;\beta_{m}=4\;\exp\left(-\frac{v+70}{18}\right)$$ (8)

$$\alpha_{h}=0.07\;\exp\left(-\frac{v+70}{20}\right),\;\beta_{h}=\frac{1}{1+\exp\left(-\frac{v+40}{10}\right)}$$ (9)

$$\alpha_{n}=\frac{0.1(v+60)}{1-\exp\left(-\frac{v+60}{10}\right)},\;\beta_{n}=0.125exp\left(-\frac{v+70}{20}\right)$$ (10)

$$\frac{dm}{dt}=\alpha_{m}(v)(1-m)-\beta_{m}(v)m$$ (11)

$$\frac{dh}{dt}=\alpha_{mh}(v)(1-h)-\beta_{h}(v)h$$ (12)

$$\frac{dn}{dt}=\alpha_{n}(v)(1-n)-\beta_{n}(v)n$$ (13)

Specifically, α and β appear as coefficients in these equations and represent transition probabilities of m, n and h gates. Na^+^ and K^+^ channels were only active when their respective gates were open. Membrane potentials depended on interaction of Na^+^, K^+^ and leakage channels.

This approach captured the induced membrane potentials caused by external injected current at the level of the soma. To improve computational efficiency and accuracy, a single-compartment soma (L = 9.6 μm, D = 9.6 μm) was constructed in NEURON (v7.5) software [13]. All parameters for the soma and Hodgkin–Huxley model are shown in Table 2 [14]. An effective action potential was simply defined as: membrane potential crossed a threshold of 30 mV in the absence of fatigue. If membrane potentials exceeded the threshold multiple times within a short period of 1 ms, it was not considered as an effective action potential because a large depolarization above threshold within a short period will cause neuronal fatigue [15].

**Table 2:** Model parameters for the Hodgkin–Huxley model and soma.

Name	Value & unit	Description
*C*_*m*_	1 μF/*cm*^2^	Membrane capacitance
${\bar{g}}_{Na}$	120 m/*cm*^2^	Sodium conductance
${\bar{g}}_{K}$	36 m/*cm*^2^	Potassium leak conductance
*g*_*l*_	0.3 m/*cm*^2^	Leak conductance
*E*_*Na*_	45 mV	Sodium reversal potential
*E*_*k*_	-82 mV	Potassium reversal potential
*E*_*leak*_	-59 mV	Leak reversal potential
*L*	9.6 μm	Length of soma
*D*	9.6 μm	Diameter of soma

### Integration of FEM and neuronal action potential model

The conversion between intracellularly injected current values *I*_*stim*_ over entire surface of soma in NEURON and current densities *J* in FEM [16] was required by an appropriate scale factor *F*:

$$F\times \frac{1}{\pi DL}\times I_{stim}(nA)=J(\frac{\mu A}{{cm}^{2}})$$ (14)

First, we found the required peak value of standard envelopes that can evoke an effective action potential in every envelope. For example, with regard to carrier frequency of 2 kHz and beat frequency of 100 Hz, the required injected current (*I*_*stim*_ = 2 *nA*, peak value) was equivalent to current density (*J* = 690 *μA*/*cm*^2^) calculated from FEM.

Second, we set output amplitude of each channel to a value that makes induced current densities at the target region half the current density that is required because the peak value of the envelope was the superposition of two channels with different frequencies have the same phase when their respective positive peak is obtained.

Third, we chose current densities in each 1 mm grid in the cross-section of the brain model from FEM. Based on temporal summation, various spatial weightings and Eq 14, current densities were integrated into certain stimulus waveforms. The stimulus waveforms, as an injected current stimulating the soma, were imported into NEURON to calculate membrane potentials and to determine whether envelopes can evoke effective action potentials. The level of neuronal activity was measured in terms of the number of action potentials generated per unit time. Therefore, the excitable region was defined as the number of action potentials generated in a complete cycle that was more than half of the maximum value (target region). In other words, the firing rate of neurons in hypo-excitable region was less than half of the beat frequency (Δ*f*). For the number of action potentials maps, collected points were linearly interpolated using MATLAB’s griddata function.

### In vitro measurement of stimulus waveforms

**Fig 3:**
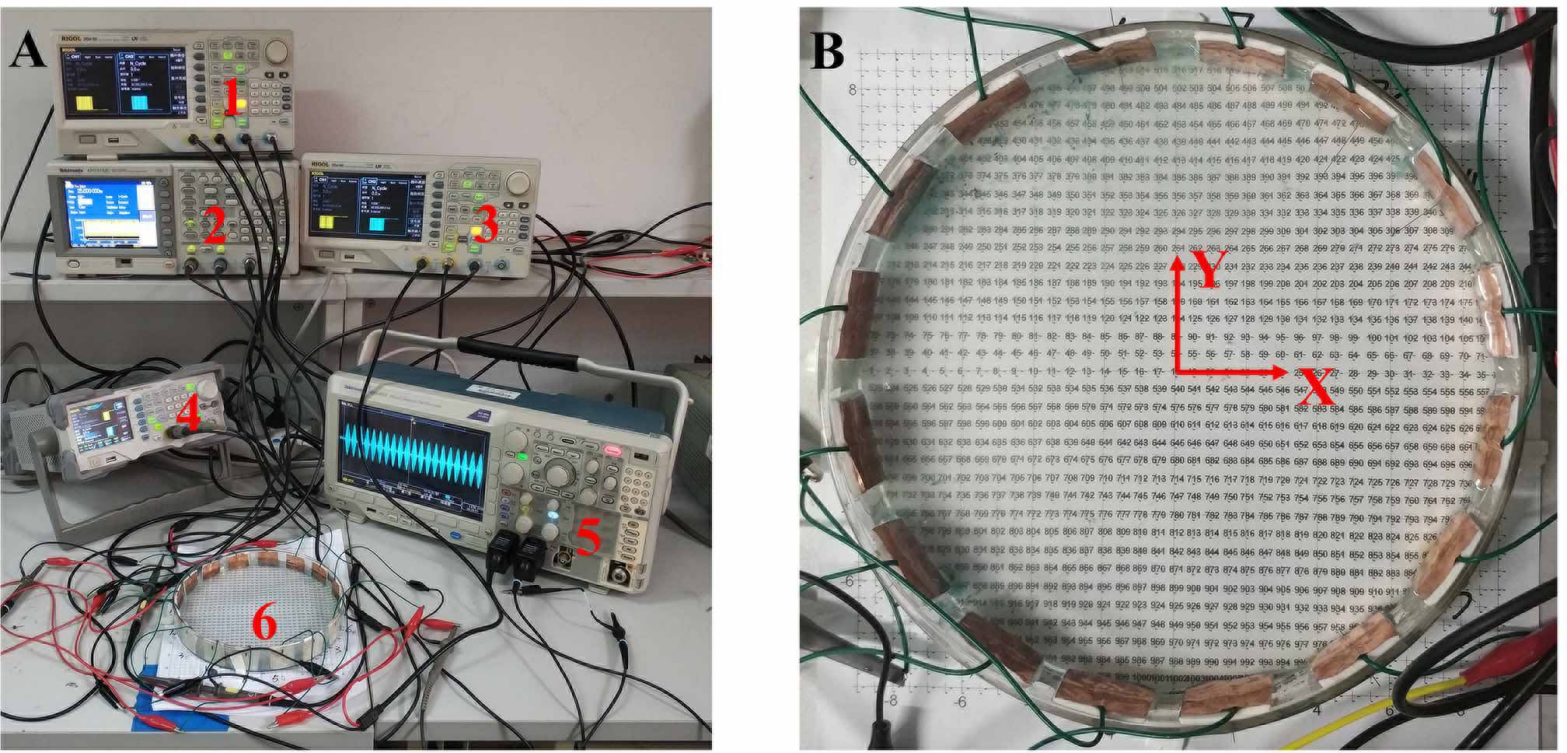
Measuring platform for the distribution of stimulus waveforms in saline solution. (A) No. 1–4 represent arbitrary waveform generators, No. 5 represents oscilloscope and No. 6 represents petri dish. (B) Petri dish with a diameter of 18cm, 5 mm grid for measuring stimulus waveforms and 16 electrodes whose arrangement and connection are the same with Fig 2B.

As illustrated in Fig 3, a petri dish with a diameter of 180 mm, was filled with 0.9% saline solution (mixture of NaCl and deionized water at temperature of 27°C) to mimic the human brain and to measure stimulus waveforms via oscilloscope (Tektronix MDO3052). Four arbitrary waveform generators (Tektronix AFG3152C, ROGOL DG1000 and ROGOL DG4162) consisted of 8 channels and were connected to multiple electrodes. Each generator had two channels and asynchronous trigger signal. Multiple electrodes, with materials of copper foil and a size of 25 mm × 25 mm, were mounted along the circumference of the petri dish (interelectrode space of 37.5 mm). The arrangement of electrodes was in accordance with simulation in FEM (Fig 2B). A needle attached to oscilloscope was inserted in the saline solution to measure stimulus waveforms every 5 mm grid. The reference of the oscilloscope was connected to the waveform generator’s ground. The oscilloscope recorded the stimulus waveforms with sampling time of 200 ms and sampling rate of 50 kHz.

Adjusting output amplitude of each channel, we calibrated stimulus waveforms in the target location to standard low frequency envelopes. After filtering out the high frequency noise (> 2500 Hz) and unit normalization, stimulus waveforms measured in each grid were then imported into NEURON to calculate the number of action potentials in two cycles (40–200 ms), which were subsequently averaged. Similarly, neuronal excitability at various points across the petri dish were linearly interpolated using MATLAB’s griddata function.

## Results

### Temporally interfering stimulation

**Fig 4:**
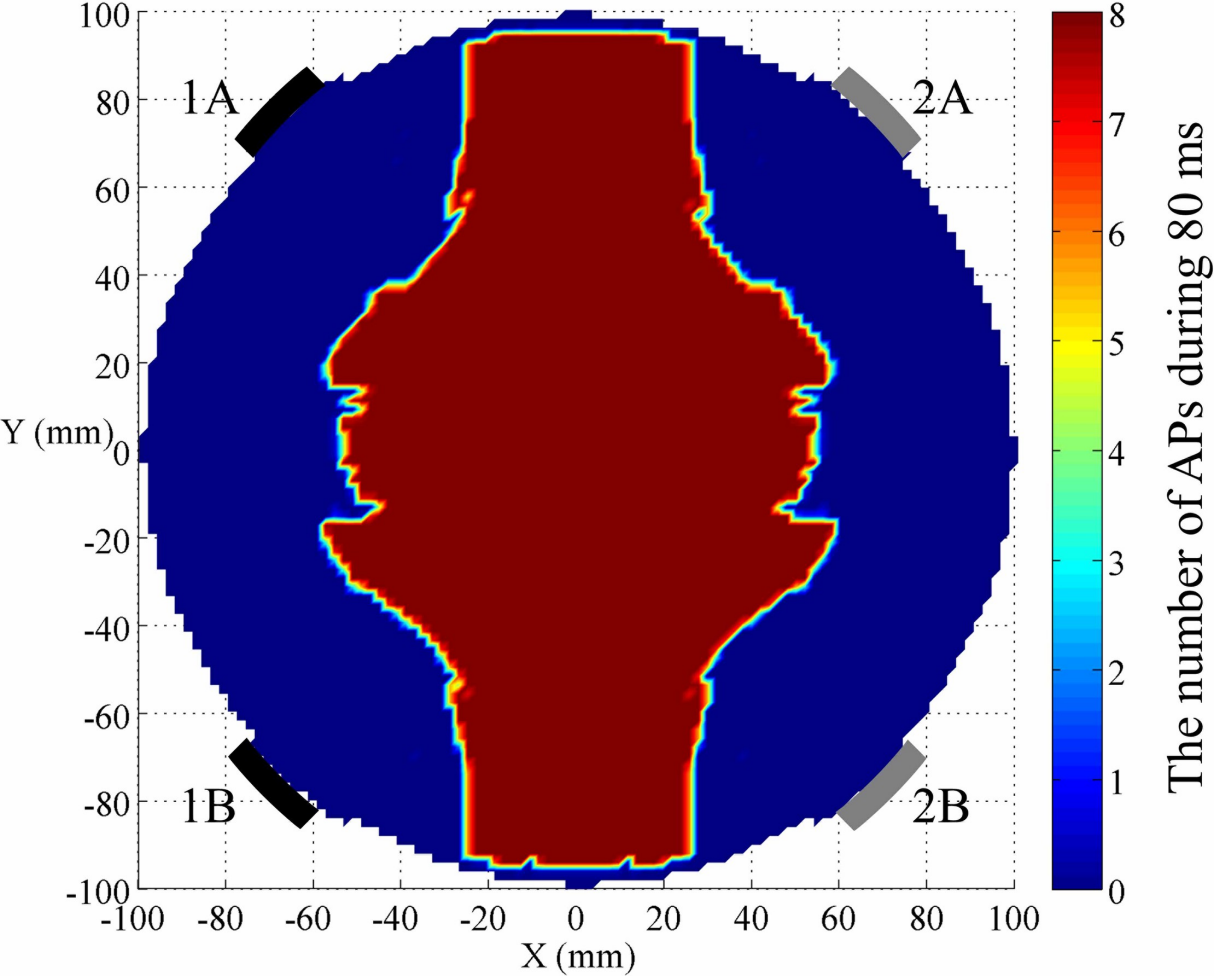
Neural excitability across the brain model based on TI stimulation [6]. Sinusoidal waveform (*f*_1_ = 2 kHz) connects the black electrode 1A (output electrode) and 1B (return electrode). Sinusoidal waveform (*f*_2_ = 2.1 kHz) connects grey electrode 2A (output electrode) and 2B (return electrode). Color bar represents the number of action potentials in a complete cycle of 80 ms.

We first repeated temporally interfering (TI) stimulation with two channels reported in the literature [6] in our simulation model combining FEM and NEURON. As illustrated in Fig 4, there were only two electrode pairs applying sinusoidal waveform with different frequencies (black: *f*_1_ = 2 *kHz*, grey: *f*_2_ = 2.1 *kHz*), and same peak value of 39 V. The basic temporally interfering electric fields caused a large area of neurons to fire. This simulation indicated that by only utilizing two channels with beat frequency of 100 Hz, spatial resolution was relatively low for the human brain model (radius of 100 mm) and needed to be improved.

### Penetration depth

We can utilize the MMS neurostimulation method to improve spatial resolution. According to the stimulus waveforms and arrangement of electrodes in Fig 1 and Fig 2B, we selected carrier frequencies of 2 kHz and 2.1 kHz, beat frequency of 100 Hz and a complete cycle of 80 ms. The amplitude of each channel was 39 V to ensure that the target region was the center of the brain model. In the central target region, the induced electric field and potential were 27.59 V/m and 19.75V, respectively. The maximum electric field (near the electrode contact) in the brain medium was 43.56 V/m.

**Fig 5:**
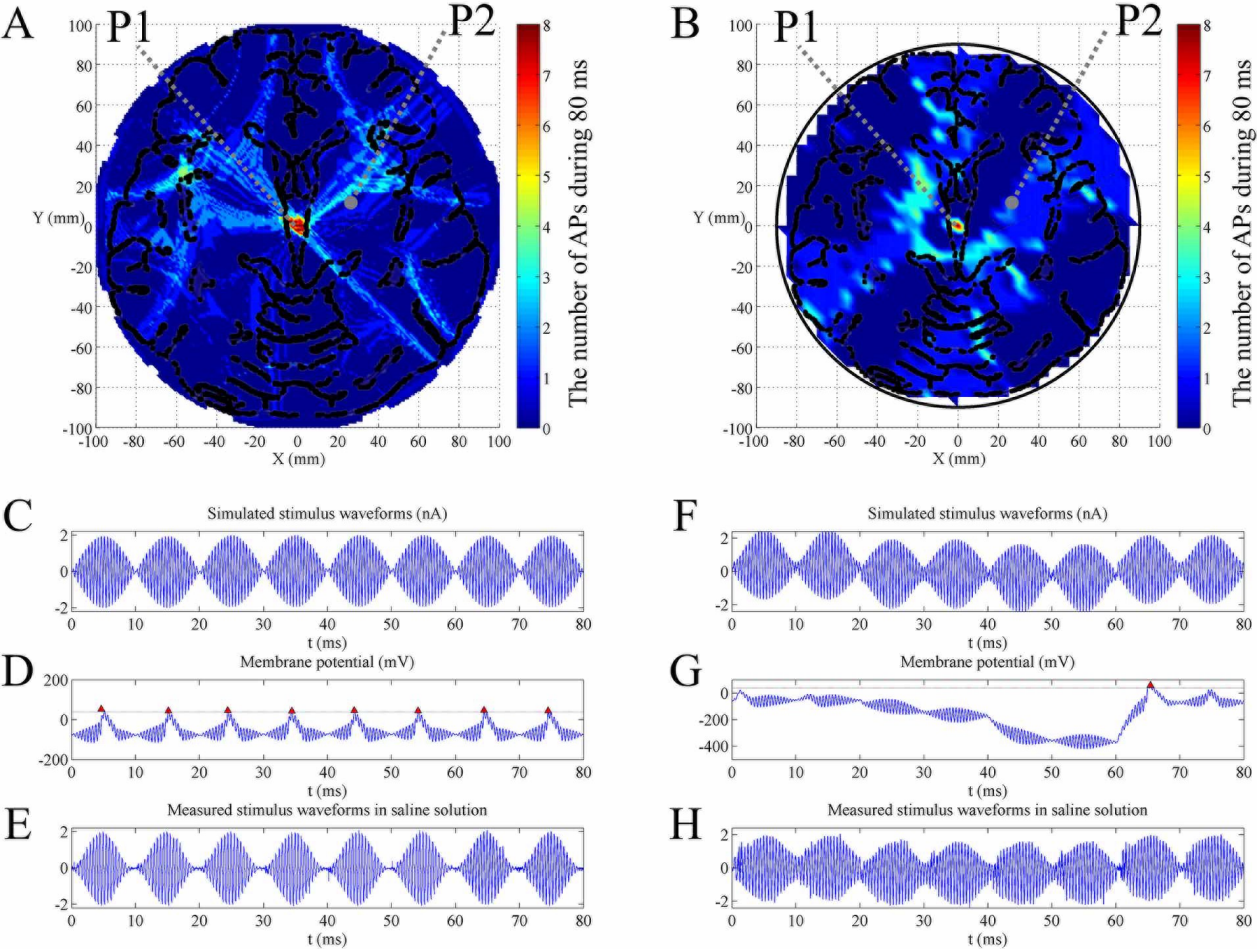
Neural excitability across the brain model when the target location was at the center of the brain based on MMS stimulation. (A) The number of action potentials map simulated in NEURON obtained from simulated stimulus waveforms. (B) The number of action potentials map simulated in NEURON based on recorded stimulus waveforms in saline solution. The arrangement of electrodes is shown in Fig 2B. The amplitude of each channel was 39 V. Locations P1 and P2 represent target region and non-target region. Color bar represents the number of action potentials in a complete cycle of 80 ms. (C-E) Target region P1 with coordinate (0 mm, 0 mm): (C) simulated stimulus waveforms from FEM (I _stim1,2,3 …8_ = 2 nA), (D) membrane potential simulated in NEURON and (E) measured stimulus waveforms in saline solution. (F-H) Non-target region P2 with coordinate (22 mm, 8 mm): (F) simulated stimulus waveforms from FEM (I_stim1_ = 1.18 nA, I_stim2_ = 0.91 nA, I_stim3_ = 0.74 nA, I_stim4_ = 1.31 nA, I_stim5_ = 0.90 nA, I_stim6_ = 1.20 nA, I_stim7_ = 1.29 nA, I_stim8_ = 0.75 nA), (G) membrane potential simulated in NEURON and (H) measured stimulus waveforms in saline solution. Red markers in membrane potential in (D) and (G) indicate effective action potentials. All stimulus waveforms and membrane potential are recorded in a complete cycle of 80 ms.

Both in simulated and measured stimulus waveforms, the target region where neurons generated the most excitability was controlled in a small central circle with MMS as demonstrated in Fig 5A and Fig 5B. The number of action potentials in non-target region was far less than in the target region. Additionally, the stimulus waveforms measured in saline solution were in accordance with results from FEM as shown in Fig 5C–5H. The trend and spatial resolution of the measured stimulus waveforms acting on neurons were in accordance with the simulated waveforms.

Within the target stimulus region P1, the center of brain, the induced current densities by each electrode pair were approximately equal. During every 10 ms as shown in Fig 5D, the soma evoked an effective action potential in response to standard low frequency envelope modulated at the frequency of 100 Hz. Every envelope’s peak value exceeded the threshold (2 nA) and had sufficient relaxation time, especially with equal positive and negative polarity, resulting in depolarization. In a complete cycle (80 ms), the effect of the stimulus waveform was approximately equivalent to a continuous 100 Hz sinusoidal wave that can elicit desired firing rate.

In the non-target region P2, the number of action potentials in a complete cycle was far less than the maximum (target region). Temporal summation of current densities with different spatial weightings produced asymmetric envelopes with unequal positive and negative polarity, as illustrated in Fig 5G. There were few or even no action potentials during a complete cycle (80 ms). In other words, firing rate (or the number of action potentials in a complete cycle) was less than half maximum value of the target region.

### Resolution

**Fig 6:**
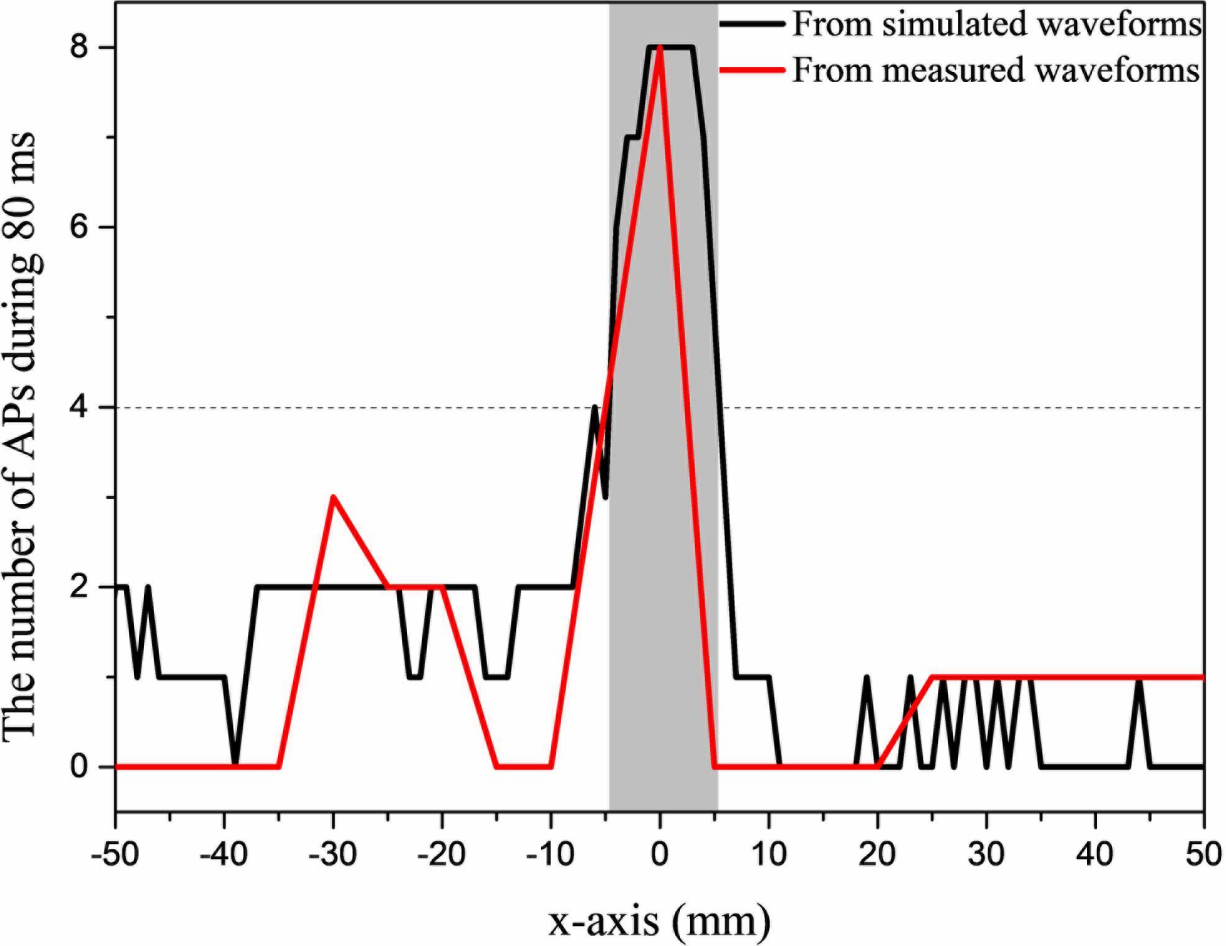
Change in the number of action potentials along x-axis when the target location was at the center of the brain. Black: the number of action potentials obtained from simulated stimulus waveforms. Red: the number of action potentials simulated in NEURON based on recorded stimulus waveforms in saline solution. Grey rectangle represents the excitable region where the number of action potentials is above half maximum value (target region). The recorded time is a complete cycle of 80 ms.

Changes in the number of action potentials along x-axis are shown in Fig 6. Deviating 5 mm away from the region of activation, the number of action potentials dropped to less than 4 (half of maximum), both in simulated and measured waveforms. Combining Figs 5 and 6, the excitable region (the grey rectangular in Fig 6) where neuronal firing rate was above half of beat frequency was controlled with a circle with a radius of 5 mm, while electrodes were 10 cm away from the target region.

### Steerability

**Fig 7:**
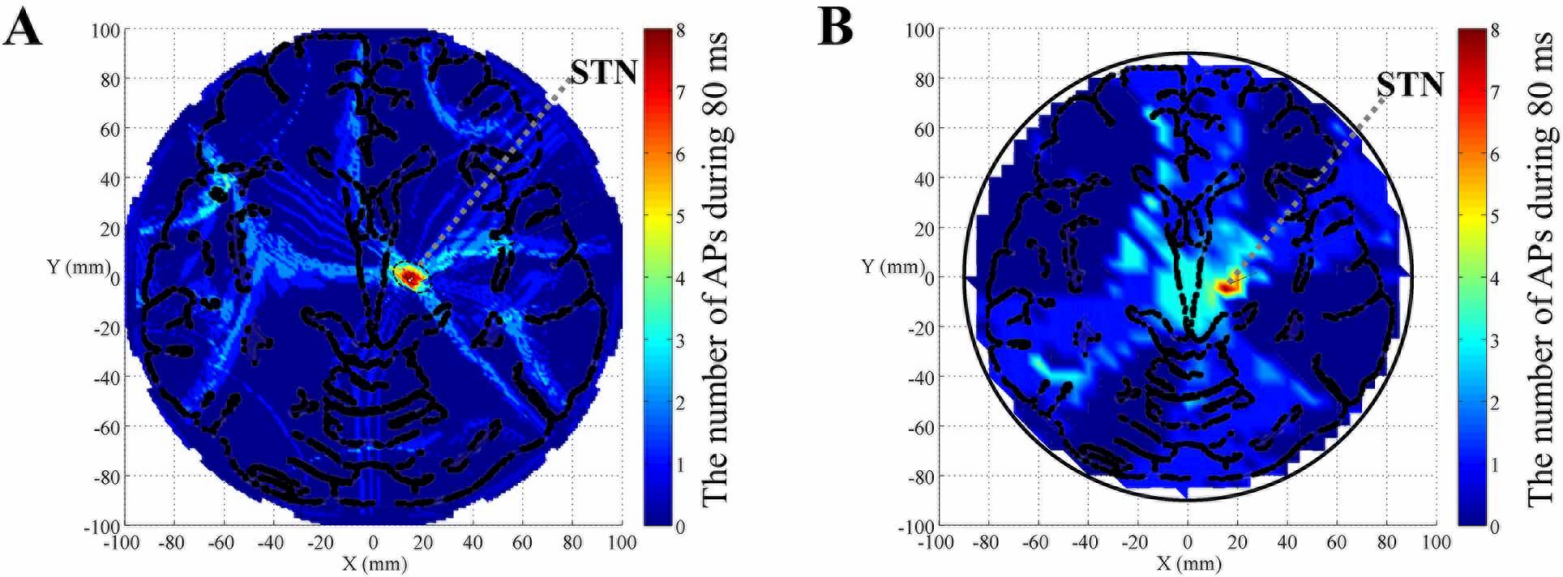
Neural excitability across the brain model when the target location was STN, based on MMS stimulation. (A) The number of action potentials map obtained from simulated stimulus waveforms. (B) The number of action potentials map simulated in NEURON based on recorded stimulus waveforms in saline solution. All stimulus waveforms and membrane potential are recorded in a complete cycle of 80 ms. The arrangement of electrodes is shown in Fig 2B. The amplitude of each channel is illustrated in Table 3.

To demonstrate our method’s steerability, we set the target region in an off-center area with coordinate (15 mm, -5 mm), consistent with the imaging position of the subthalamic nucleus (STN) on the axial plane [17]. Maintaining the original electrode arrangement (as shown in Fig 2B), our method just adjusted each channel’s outputting amplitude to a certain value respectively until current densities were equal to each other in STN. The voltage amplitude applied to each channel is shown in Table 3. As demonstrated in Fig 7, the most excitable region was the STN. Importantly, MMS still maintained its precision both in simulation and in measurement.

**Table 3:** The voltage amplitude applied to each channel when the target location was STN.

Channel	CH1	CH2	CH3	CH4	CH5	CH6	CH7	CH8
Amplitude (V)	33.5	33	45.2	32.8	42.2	33.6	42.2	34

## Discussion

Based on temporally interfering (TI) electric fields [6] with different frequencies and computational model, we introduce a noninvasive multiple modulation synthesis method that can effectively stimulate deep neurons at the desired firing rate (beat frequency) with higher spatial precision compared to TI stimulation. The excitable region is determined not only by current density, similar to the convention transcranial electrical stimulation, but also by specific characteristics of the waveforms. Compared to conventional transcranial electrical stimulation, MMS method can stimulate deeper neurons while keeping high spatial precision.

Each channel elicits current density with different spatial weightings across the brain model due to attenuated trends of current density in less conductive tissue medium. Only in the target region, stimulus waveforms are superposed by equal amplitude, resulting in symmetric, standard, and low-frequency envelopes. Once deviated from the target region, the current densities generated by each channel are no longer equal and their spatial weightings are different, resulting in asymmetric waveforms. The reasons why asymmetric waveforms cannot elicit action potentials are attributed to the following two points. First, the positive and negative polarity of the envelope are not equal, resulting in hyperpolarization instead of depolarization in a certain time slot. Second, the envelopes are more inclined to high-frequency properties without sufficient relaxation time and cannot recruit neuronal firing.

We utilize single-sign and kilohertz-frequency waveforms to improve spatial resolution, however, some safety considerations should be considered. On the one hand, monophasic waveforms may cause more tissue damage than biphasic waveforms [18]. For example, in close to electrodes (electrode 1A in Fig 2B), the stimulus waveforms accumulate much positive charge during the first time slot (0–40 ms). However, during the second timeslot (40–80 ms), the negative electrode (electrode 7B in Fig 2B) will bring negative charge. Therefore, charge density achieves a balance between positive and negative polarity, and this is approximately equivalent to biphasic waveforms in a complete cycle (80 ms). On the other hand, kilohertz-frequency current may require relatively high amplitude (30–50 V) to activate deep neurons. The amplitude of current is similar to the motor threshold of kilohertz-frequency current [19].

We define an excitable region as its firing rate that is higher than half of the beat frequency. Inevitably, switching between time division (on and off) will induce several action potentials in hypo-excitable regions because it brings discontinuous stimulus waveforms with different polarity trends. As illustrated in Fig 5F and Fig 5G, for example, during 40–60 ms, the overall polarity trend of stimulus envelopes with more negative polarity cause a large hyperpolarization and de-inactivation of voltage-gated sodium channels. During the next period (60–70 ms), however, an action potential is induced due to anode break excitation [20], which cause a drop in the threshold required for action potential. The existence of several action potentials (13–38 Hz) in the non-target region is an inherent limitation and trade-off between depth and focality, which are in accordance with recent findings of transcranial electrical stimulation [21]. However, in non-target regions where neurons are hypo-excitable, not all stimulus envelopes can be equivalent to 100 Hz sine or pulse waves that elicit the desired firing rate.

In the FEM computational model, the simplified multi-layered brain model is not as accurate as models established by MR scans. Inevitably, current density is influenced by thickness, shape and dielectric parameter of tissue as well as individual variability. In the subsequent optimization, more precise brain models [22] with accurate dielectric parameter will be introduced to further determine the target region, spatial resolution and optimize the arrangement of electrodes. When we steer the target region, we should adjust and calibrate output amplitude of each channel until current densities by various channels are equal in the desired target region. Relatively far from the target region, electrode output amplitude is greater than electrodes with a smaller distance from the target region. Precise calibration of each channel ensures that only in the target region, the stimulus waveforms are symmetric and standard envelopes with superposition of equal current densities. Spatial resolution may be further improved by optimizing arrangement and shape of electrodes, the number of time division group and so on.

We only establish single-compartment neurons with three fundamental active ion channels instead of considering neural networks and complicated morphologies. Although neurons have different properties, some are common. First, neurons have basic characteristics of a low-pass filter and high-frequency fatigue that prevents neuronal electrical activity caused by kilohertz-oscillating electric fields. Second, positive currents depolarize the membrane while negative currents hyperpolarize it [16]. Single-compartment neuronal models have led to a quantitative understanding of stimulus waveforms via ion channels. In future work, a more detailed neuronal network, including the concomitantly modulation of excitatory and inhibitory neurons [23], high-frequency electrical block [24] will be adopted to establish a complete computational neuron model. In addition, live experiments will be considered to further verify its clinical therapeutic effects, including long-term effects, individual differences in patients, the optimal frequency (carrier frequency, envelope frequency and the frequency of stimulation session) and actual spatial resolution.

## Conclusion

In conclusion, we propose a noninvasive method that entrains deep neurons at desired frequency, and it approaches high spatial resolution via finite element analysis, action potential modeling and measuring stimulus waveforms in saline solution. We demonstrated that it can control excitable region of deep neurons at the desired firing rate with higher spatial resolution compared to TI stimulation. Our proposed method that utilizes multiple (time-division, frequency & polarity) modulation synthesis (MMS) stimulus waveforms will provide a basic scheme for noninvasive deep neurostimulation as a potential alternative to implanted electrodes. Moreover, our methods support the further development of designing special stimulus waveforms.

## Supporting information

S1 Table The frequency and polarity of various channels in a complete cycle of 80 ms based on MMS stimulation. (DOCX)

S1 File Code for finite element method. (M)

S2 File Code for NEURON. (ZIP)
