# Supplementary material for: A multiple modulation synthesis method with high spatial resolution for noninvasive neurostimulation
Source: PLoS One. 2019 Jun 20;14(6):e0218293. doi: 10.1371/journal.pone.0218293 (PMC6586313; doi:10.1371/journal.pone.0218293)
Supplement: S1 Table — (DOCX) [file pone.0218293.s001.docx]

| Period (ms) | Frequency / Polarity (positive: +, negative: -) | | | |
| --- | --- | --- | --- | --- |
|  | (T+0, T+20) | (T+20, T+40) | (T+40, T+60) | (T+60, T+80) |
| CH 1 | 2 kHz / + | 2.1 kHz / + | / | / |
| CH 2 | 2 kHz / - | 2 kHz / - | / | / |
| CH 3 | 2.1 kHz / - | 2.1 kHz / - | / | / |
| CH 4 | 2.1 kHz / + | 2 kHz / + | / | / |
| CH 5 | / | / | 2 kHz / + | 2.1 kHz / + |
| CH 6 | / | / | 2 kHz / - | 2 kHz / - |
| CH 7 | / | / | 2.1 kHz / - | 2.1 kHz / - |
| CH 8 | / | / | 2.1 kHz / + | 2 kHz / + |
